# Hybrid Modified *K*-Means with C4.5 for Intrusion Detection Systems in Multiagent Systems

**DOI:** 10.1155/2015/294761

**Published:** 2015-06-15

**Authors:** Wathiq Laftah Al-Yaseen, Zulaiha Ali Othman, Mohd Zakree Ahmad Nazri

**Affiliations:** ^1^Data Mining and Optimization Research Group (DMO), Centre for Artificial Intelligence Technology (CAIT), School of Computer Science, Faculty of Information Science and Technology, Universiti Kebangsaan Malaysia (UKM), 43600 Bandar Baru Bangi, Malaysia; ^2^Al-Furat Al-Awsat Technical University, Iraq

## Abstract

Presently, the processing time and performance of intrusion detection systems are of great importance due to the increased speed of traffic data networks and a growing number of attacks on networks and computers. Several approaches have been proposed to address this issue, including hybridizing with several algorithms. However, this paper aims at proposing a hybrid of modified *K*-means with C4.5 intrusion detection system in a multiagent system (MAS-IDS). The MAS-IDS consists of three agents, namely, coordinator, analysis, and communication agent. The basic concept underpinning the utilized MAS is dividing the large captured network dataset into a number of subsets and distributing these to a number of agents depending on the data network size and core CPU availability. KDD Cup 1999 dataset is used for evaluation. The proposed hybrid modified *K*-means with C4.5 classification in MAS is developed in JADE platform. The results show that compared to the current methods, the MAS-IDS reduces the IDS processing time by up to 70%, while improving the detection accuracy.

## 1. Introduction

With the growing demand for the services provided by networks, the availability, confidentiality, and integrity of critical information has become increasingly at risk from misuse [1–3]. Firewall systems alone provide insufficient protection from unwanted access to this important information due to their inability to protect networks from intruders using open ports [4–6]. Intrusion Detection System (IDS) is one of the system security infrastructures attempting to detect malicious activities, such as denial of service attacks and port scans, by monitoring and analyzing events occurring on networks and computers [1, 7]. In terms of intrusion detection, IDS can be classified as either host-based or network-based. The host-based IDS (HIDS) observes the behavior and state of the computer activities and detects the programs that can gain access to resources. On the other hand, the network-based IDS (NIDS) is monitoring the network traffic (traffic volume, service ports, IP addresses, and protocol usage) and analyzes it to identify suspicious activities [8–10]. In general, IDS can be implemented using two approaches: rule-based detection and anomaly-based detection [1, 10]. Rule-based detection (also known as misuse or signature-based detection) searches for specific signature patterns previously stored in the rules database. Snort is one of the popular approaches used in its work to detect intrusions based on rules [11]. The disadvantage of rule-based detection is inability to detect new attacks, as these have no signatures in the database [4]. Thus, rule-based detection will increase the percentage of false negative results. On the other hand, the anomaly-based detection approach constructs models of all normal activities through the observed data and then alerts of any behavior or activity that deviates from this model [12]. The main advantage of anomaly-based detection stems from its capability to detect novel attacks, which are different from the already learned attacks. However, its drawback is the increased likelihood of classifying normal behavior as attacks, thus increasing the false positive rate [13].

Most researchers studying these issues focused on the accuracy of detection attacks by using different methods, such as neural networks [14, 15], fuzzy logic [16, 17], and machine learning [18–20], thus dedicating less attention to the processing time required to detect attacks. However, processing speed is becoming more important, due to the increasing network traffic and the need to achieve the objective of intrusion detection systems as real time systems.

One of the popular methods for reducing processing time is distributed artificial intelligence (DAI) that merges artificial intelligence with distributed computing [21]. Multiagent system (MAS) is one of the DAI branches. Several authors have proposed agents aimed at improving the IDS performance. For example, JAM [22] used metalearning distributed data mining to build classifiers at difference nodes for data analysis. This system has a manager responsible for coordinating simultaneous execution of classifiers by agents. It subsequently combines the results of all classifiers by using one of the Meta learning techniques. On the other hand, DIDMA [23] uses two types of agents: (1) static agent responsible for collecting the information about attacks from its host and (2) mobile agent responsible for gathering from static agents the information pertaining to new attacks on the system. However, in these works, agents are applied in IDS as a conceptual idea, without demonstrating their performance.

Over the years, various machine learning techniques have been proposed, with their authors claiming that their performance is best suited for IDS [10]. *K*-means and decision trees are among these techniques that are used widely in designing IDS. *K*-means is used to cluster the data to find the meaningful structures or patterns in a collection of unlabeled data, so that the instances in the same cluster are similar, while the instances from different clusters are different from each other. Several extant studies [24–29] presented *K*-means as a single algorithm for clustering the IDS data into a set of clusters representing normal processes and attacks. Furthermore, different authors [30–34] have presented combined methods, depending on *K*-means and other techniques, which were used to build the model of IDS. On the other hand, C4.5 is used to build a tree structure of attack signatures, as well as constructing the tree structure of normal behaviors. This approach depends on maximum information gain in the feature selection criterion and minimal information split into building the tree structure. In some approaches, C4.5 is used as a single model to construct misuse detection only [35, 36], while other researchers combined C4.5 with other techniques to build IDS as a layered model [37]. Finally, in some studies [38–41] C4.5 was evaluated and compared with other techniques in order to demonstrate its performance. However, combining *K*-means with decision trees has resulted in good IDS accuracy. For example, in a recent study [30], C4.5 technique was used with *K*-means to design a supervised anomaly detection system, while other research group [42] used ID3 combined with *K*-means in a novel supervised anomaly detection approach.

This study proposes a hybrid modified *K*-means with C4.5 algorithms to build IDS in a multiagent system environment. The aim of this approach is to improve the anomaly-based detection accuracy, while the MAS is required to reduce the IDS processing time. In the proposed design, MAS utilizes three types of agents: coordinator, analysis, and communication agent. The coordinator agent is responsible for building trees of the training dataset through the use of modified *K*-means to cluster the data into a number of clusters. It subsequently uses the C4.5 technique to build the tree for each cluster. The resulting trees exhibit high efficiency in classifying the testing data because each tree is built from similar instances as attributes. Thus, it is possible to discriminate between classes with high accuracy. Moreover, these trees will reduce the processing time, as search is performed on a smaller tree. The second task of the coordinating agent is dividing the testing dataset into a number of subsets. Moreover, this agent will send every subset of testing data with the training trees to one of the analysis agents responsible for analyzing it. Lastly, the coordinator agent combines the results yielded by the analysis agents in order to obtain the final results. On the other hand, the analysis agent is responsible for analyzing the data received from the coordinator agent. It is using the closest decision tree to classify each instance of the dataset into the appropriate class. Finally, the communication agent is responsible for transferring the data and results between coordinator agents and analysis agents. The modification of *K*-means is the method adopted for choosing the initial centroids of clusters. This work will significantly reduce the processing time, thus increasing the IDS efficiency. KDD Cup 1999 dataset is used to evaluate the performance of the proposed system, and JADE platform is used for its implementation.

The remainder of this paper is organized as follows.  Section 2 provides a brief review of the related work on *K*-means, C4.5, and multiagent systems with IDS. The proposed system is described in Section 3, while Section 4 presents experimental results, in order to demonstrate the proposed system performance. The concluding remarks are given in Section 5.

## 2. Related Work

This section provides a detailed description of the role of multiagent system in IDS and discusses the role of *K*-means and C4.5 algorithms in building the IDS models. Extant studies have confirmed that C4.5 technique can achieve better classification performance. In addition, *K*-means has high ability to group the data into clusters, where the instances in same cluster have high similarity.

### 2.1. Multiagent Systems (MAS)

Various multiagent systems (MASs) for IDS have been proposed [43–47]. Dasgupta et al. [43], for example, developed the Cougaar framework and presented hierarchical architecture consisting of four different agents (manager, monitor, decision, and action agent). The authors used intelligent decision support modules, such as fuzzy inference system, to detect anomalies at the packet, process, user, and system level. This work, however, fails to explicate how multiple security nodes of CIDS should be organized when large numbers are needed to protect many hosts in larger networks. Moreover, the authors do not discuss how and what information the manager agents should share. The architecture of multiagent, flow-based IDS was developed in a different study [44], where the concept of reputation system was used to permit agents to find nodes that are most effective for classifying malicious network activity. Zhu et al. [45] presented MAIIDS using more than one technique, such as neural network, association rules for learning agents, and generating rules for decision agents, to detect the audit data according to these rules and respond to them. The experimental results indicate that their system has very high self-adapting ability, intelligence, and expansibility. El Ajjouri et al. [46] presented architecture based on adding a learning feature, whereby abnormal behaviors correspond to unknown malicious patterns. This architecture first detects new attacks using the agent responsible for detecting the new behavior, after which it updates the basic attack patterns. This agent used case-based reasoning (CBR) technique as the attack detection method. Yang et al. [47] presented distributed agent model dependent on artificial immune systems (AIS) for building IDS. This system takes the features of AIS, such as self-adapting, self-learning, self-organizing, parallel processing, and distributed coordinating. Although this work includes a section on empirical findings, the authors do not provide any details about the experiments they have conducted. Therefore, it is not possible to ascertain the significance of their results. As can be seen from above, none of the authors of extant works on agent-based IDS discussed or presented the results of processing time clearly. This shortcoming is addressed in the present study, where one of the measurements that evaluate the performance of IDS based on MAS requires computing the IDS processing time.

### 2.2. *K*-Means Algorithm-Based IDS

*K*-means algorithm is the method that clusters groups of objects into *k* disjoint clusters based on their attributes [48]. The objects in the same clusters are similar, while those from different clusters differ from one another. This algorithm uses one of the similarity measures to compute the distance between two objects. The measure most commonly used by *K*-means is the Euclidean distance. The advantage of *K*-means is its flexibility in dealing with large datasets [25], with the time complexity *O*(*tkn*), where *t* represents the number of iteration times, *k* denotes the number of clusters, and *n* is the number of dataset instances. However, the main disadvantage of the *K*-means algorithm is the need to find the best number of clusters *k*. In addition, it is sensitive to the isolated dataset instances [25], and the algorithm converges finitely to local minima. Consequently, the initial centroids of clusters significantly affect the *K*-means algorithm output. The pseudocode of standard *K*-means is shown in Pseudocode 1 [49], where

(1) $$\begin{array}{c} \left\|\;x-y\;\right\|=\sqrt{\overset{\text{no Attributes}}{\underset{i=1}{\sum }}{\left(x_{i}-y_{i}\right)}^{2}}. \end{array}$$

In the past, many ideas were intended to improve the performance of *K*-means. Most of these methods aimed at improving the method used for selecting the initial centroids of clusters. For example, Ball and Hall [50] adopted the centroid of the dataset as the first centroid; that is, *X*′ = 1/*N∗*∑_*j*=1_^*N*^ *x*_*j*_, before choosing the remaining centroids in arbitrary fashion, if the distance between them and previously selected centroids is greater than the threshold, until *k* centroids are obtained. Maximin method developed by Katsavounidis et al. [51], on the other hand, chooses the first centroid arbitrarily, while the subsequent centroids (*k* − 1) are chosen as instances that have the greatest minimum-distance with respect to the previously selected centroids. Al-Daoud's variance-based method [52] sorts the instances of data depending on the variance in attributes and then partitions them into *k* groups with the same dimension (the medians of these groups are chosen as centroids). The *k*-means++ method [53] combines 2th MacQueen with Maximin method to select the first centroid randomly and the *i*th (*i* ∈ {2,3,…, *k*}) centroid is chosen as an instance with probability md(instance′)^2^/∑_*j*=1_^*N*^md(instance_*j*_)^2^, where md(*x*) denotes the minimum-distance from the previously selected centroids. Erisoglu et al. [54] proposed a method that first chooses two main vectors representing the best dataset distribution, before computing the centroid of the dataset as a mean of these two vectors. The first cluster centroid is the instance with the longest Euclidean distance from the centroid of the dataset, while the *i*th cluster centroid is the instance with the maximum combined distance from the previous (*i* − 1) cluster centroids.

### 2.3. Hybrid *K*-Means-Based IDS

The best possible high detection rate and low false alarm rate can be achieved by using hybrid approaches for IDS. Hybrid *K*-means, combined with other techniques, played an important role in this field. Xiao et al. [31] proposed a *K*-means algorithm based on PSO for network anomaly detection. The authors used PSO to solve the problem of local convergence minimum of *K*-means, capitalizing on the PSO's global search ability. Experimental results with a KDD dataset demonstrate that the proposed method is effective in dealing with large datasets and achieves a satisfactory detection rate. Yongzhong et al. [55] also proposed a similar PSO-*K*-means hybrid system. Muda et al. [32] proposed a hybrid learning approach through a combination of *K*-means clustering and Naïve Bayes classification. The authors clustered all data into the corresponding group before applying a classifier for classification purpose. Their results show that the proposed approach achieved reasonable false alarm rate. A clustering algorithm that uses SOM and *K*-means for intrusion detection was proposed by Wang et al. [33]. When the SOM finishes its training process, *K*-means is adopted to refine the weights obtained by training. In addition, once SOM completes cluster formation, *K*-means is applied to refine the final clustering results. Chandrasekhar and Raghuveer [34] proposed a new approach based on fuzzy neural network and support vector machine to improve the IDS detection rate. Here, *K*-means clustering was first applied to generate different training data subsets.

However, Muniyandi et al. [30] proposed an anomaly detection method using *K*-means combined with C4.5 for classifying anomalous and normal activities. In this approach, *K*-means clustering is used initially to partition the training dataset into *k* clusters using Euclidean distance. Then, the decision tree is built for each cluster using the C4.5 technique and the rules created by the decision tree are used to detect intrusion events. The testing phase is implemented through two steps. In the first step, the Euclidean distance is computed for every testing instance, before finding the closest cluster. Therefore, the decision tree corresponding to the closest cluster is selected to detect the class of the instance. In this work, *K*-means still have some shortcomings, as the clustering output mostly depends on the selection of the initial centroids of clusters. In addition, the number of clusters *k* needs to be given in advance. Moreover, the resulting clusters do not include all the possibilities of class instances.

## 3. Proposed Hybrid Modified *K*-Means with C4.5 in MAS-IDS

**Figure 1:**
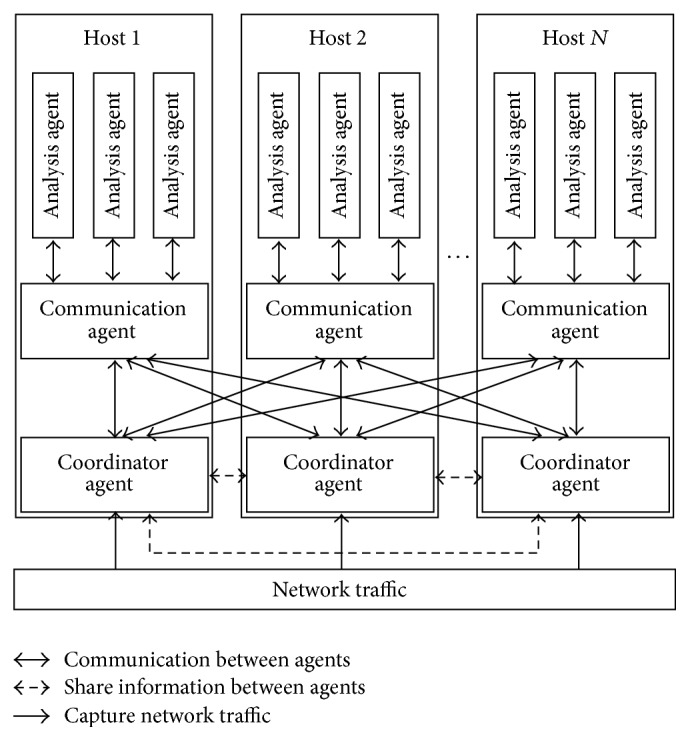
The MAS-IDS architecture.

The proposed MAS-IDS uses three agents that are responsible for achieving the IDS goals: coordinator, analysis, and communication agent. The MAS-IDS system is shows in Figure 1. The details of the proposed system are elaborated on in the next sections.

### 3.1. Multiagent System-Based Intrusion Detection System (MAS-IDS)

#### 3.1.1. Coordinator Agent

The deliberative coordinator agent uses a training dataset to train the hybrid system through constructed clusters by using modified *K*-means. It subsequently applies the C4.5 technique on each cluster to build the decision trees that will be used in the testing phase. On the other hand, the coordinator agent receives and divides the gathering traffic data network into a number of subsets by applying (2). Therefore, it sends these subsets with the trees and centroids of clusters to the analysis agents in the other hosts by using communication agents. At the same time, the coordinator agent has information about all the hosts of the system environment, where each host periodically sends the number of its cores that are presently not busy to the coordinator agent. The scenario in which the coordinator agent operates can be summarized in the following steps.

(1) Read the training dataset.
(2) Call modified *K*-means (Pseudocode  2) to cluster the training dataset into a set of clusters.
(3) Build the tree for each cluster by using the C4.5 technique.
(4) Send the trees and centroids of clusters to static agents in the other hosts.
(5) Capture traffic network data packets.
(6) Specify the number of core CPUs in hosts of the system that are presently not busy (assume *n*).
(7) Divide the captured data into *n* subsets; refer to (2).
(8) Create *n* analysis agents in the other hosts; refer to (3).
(9) Send the *n* data subsets to the analysis agents by using the communication agent.
(10) Wait until all analysis agents finish analyzing data.
(11) Combine the results yielded by the analysis agents using (4):
  (2) $$\begin{array}{c} \text{Data set}=\left\{\;S_{1},S_{2},\ldots ,S_{j},\ldots ,S_{n}\;\right\} \end{array}$$

subject to

 
*S*_*j*_ = {set  of  Instances ∈ Data set:  each  Instance ∉ *S*_*i*_},
 ∀*i* = 1,…, *n*, and *i* ≠ *j*,
 |*S*_*j*_| = |Data  set|/*n*, *j* = 1,…, *n*,

where *n* ≤ core CPUs available in the system.

#### 3.1.2. Analysis Agent

A set of reactive analysis agents is created in the other hosts within the system environment by using (3), where the number of analysis agents is equal to the number of subsets resulting from the splitting process. Each analysis agent receives one subset of testing data, along with the centroids of clusters and decision trees that have been created in the training phase by the coordinator agent. In fact, the coordinator agent sends a message about the number of agents needed to the deliberative agent resident in each host. Thus, the resident agent creates these analysis agents. Unfortunately, in JADE, if the coordinator agent is creating the analysis agents directly in the other hosts, the analysis agents are logically created in these hosts. However, physically, these agents are created in the host of the coordinator agent. Consequently, the analysis agents will be using same the core CPU and memory as the coordinator agent host. Each analysis agent is running as a thread by using one of the core CPUs on that host [56]. Hence, if this host has four cores, it can simultaneously run four threads of agents in parallel

(3) ∀Sj,  create  AAj∈Hosti⟶analysisSj,AAj,j=1,…,n;  1≤i≤m

subject to

 number of AA in Host_*i*_ ≤ number of core CPUs in Host_*i*_,

where AA_*j*_ represents the analysis agent *j*, analysis (*S*_*j*_, AA_*j*_) represents the analysis function used to analyze subset *S*_*j*_ by analysis Agent AA_*j*_, and *m* represents the number of hosts available in the system environment.

In the analysis agent, each instance is first tested with the closest centroid of clusters, after which the decision tree corresponding to this centroid is used to determine the type of instance. If the instance attributes do not match any class from the decision tree, this instance is treated as attack and the decision trees are updated with the data pertaining to this attack, to assist with future detection. The scenario in which the analysis agent operates is as follows.

1. Receive the subset data, centroids of clusters, and trees from the communication agent.
2. Call the pseudocode (Pseudocodes 3 and 4) to analyze the data.
3. Return the results to the coordinator agent by using the communication agent.

Finally, the coordinator agent combines all the results produced by the analysis agents by using (4) to provide the final results to the system administrator. At this time, the system administrator will raise an alert to deal with this situation

(4) Normal  instances=⋃i=1nNormalAAi,Attack  instances=⋃i=1nAttackAAi.

#### 3.1.3. Communication Agent

The communication agent is responsible for transferring data and results between agents. The scenario in which the communication agent operates is presented through the following steps.

1. Receive datasets, centroids of clusters, and decision trees from the coordinator agent.
2. Move the above from the coordinator agent host to the analysis agent host.
3. Give the dataset, centroids of clusters, and decision trees to the analysis agent.
4. Receive the results from the analysis agent.
5. Move the results from the analysis agent host to the coordinator agent host.
6. Provide the results to the coordinator agent.

### 3.2. Modified *K*-Means Algorithm

The main advantage of modified *K*-means that distinguishes it from other adjusted *K*-means in the extant literature is its ability to consider all possible eventualities by treating all the divergent points in the dataset as initial centroids of clusters, rather than selecting a specific set of initial centroids randomly, as is typically done. In other words, modified *K*-means constructs clusters with all the cases characterized by significant differences among instances. Thus, modified *K*-means will distribute the dataset instances to convenient clusters with best accuracy. However, unlike other adjusted *K*-means, in the modified *K*-means approach, determining the number of clusters *k* is not required, as this is done dynamically. The main difference between the modified and the standard *K*-means is in the selection of initial centroids of clusters, as shown in the following steps.

1. Select the first centroid of the cluster as the first instance of the dataset.
2. Select the instance with the distance from all the previously selected centroids greater than the specified threshold (best threshold = 4000, first experiment) as the next centroid.
3. Repeat Step (2) to reach to the end of the dataset.
4. Apply the other steps of standard *K*-means on the selected initial centroid of clusters.

Pseudocode  2 shows the pseudocode of modified *K*-means. Our modification of *K*-means is evident in Steps (1) and (2) of the pseudocode.

### 3.3. C4.5 Algorithm

After distributing the training dataset instances among the clusters by using modified *K*-means, the standard C4.5 technique developed by Quinlan [57] is used to build the trees from clusters, whereby C4.5 builds tree for each cluster. More details and the pseudocode of C4.5 can been found elsewhere [58, 59].

### 3.4. Testing Phase

This phase is implemented by the analysis agent and is executed in two stages to test the traffic data network. In the first stage, the closest centroid of testing instance is chosen (the pseudocode of this stage is shown in Pseudocodes 3). In the second stage, the subtree corresponding to the centroid chosen in the first stage is implemented in order to test the instance and identify the appropriate class for this instance. The pseudocode of the second stage is shown in Pseudocodes 4.

## 4. Experimental Setup and Analysis

We used the benchmark KDD Cup 1999 [60] to evaluate the MAS-IDS performance. In most of the previous works in this field, the authors used cross-validation, such as 10-fold, for evaluation. Cross-validation was based on using the same classes of training data without adding new classes in the testing stage. Thus, these works could achieve high performance in terms of accuracy and detection rate. On the other hand, the strength of IDS stems from its ability to detect unknown attacks (new attacks). The KDD Cup 1999 dataset consists of two datasets, 10% KDDCUP dataset (used for training) and Corrected dataset (employed in testing). More details about KDD Cup 1999 can be found in extant literature [61]. Among the available performance measures, accuracy (Acc), detection rate (DR), and false alarm rate (FAR) are most popular when aiming to evaluate the MAS-IDS performance.

(5) Acc=TP+TNTP+TN+FP+FN,DR=TPTP+FN,FAR=FPTN+FP.

The computers used to implement the experiments are equipped with Core-i7 3.40 GHz, with 8 core CPUs and 6 GB RAM. The OS is Windows 7 professional 64 bits. The experiment was conducted in JADE platform and was implemented using JAVA programming.

Table 1 shows the details of datasets used to evaluate the MAS-IDS performance, along with the conventional method (hybrid standard *K*-means with C4.5) and other techniques. It should be noted that training datasets (trainDS1, trainDS2, trainDS3, and trainDS4) were generated randomly from 10% KDDCUP dataset, while testing datasets (testDS1, testDS2, testDS3, and testDS4) were generated randomly from Corrected dataset.

The preprocessing for the symbolic attributes is achieved. The three symbolic attributes are protocol, service, and flag that convert to numeric values, such as protocol attribute. The three values* tcp*,* udp*, and* icmp* are converted to 1, 2, and 3, respectively, and the same approach is adopted for the remaining attributes.

In this study, three experiments were carried out. In the first experiment, the best value of the threshold was computed, while the MAS-IDS performance was evaluated in the second experiment by comparing the results yielded by MAS-IDS with those obtained through the conventional method and other techniques available in Weka and Matlab. In the third experiment, we compared the processing time required by MAS-IDS with that of hybrid modified *K*-means with C4.5 in nonagent environment.

### 4.1. Identifying the Best Threshold for Modified *K*-Means

**Figure 2:**
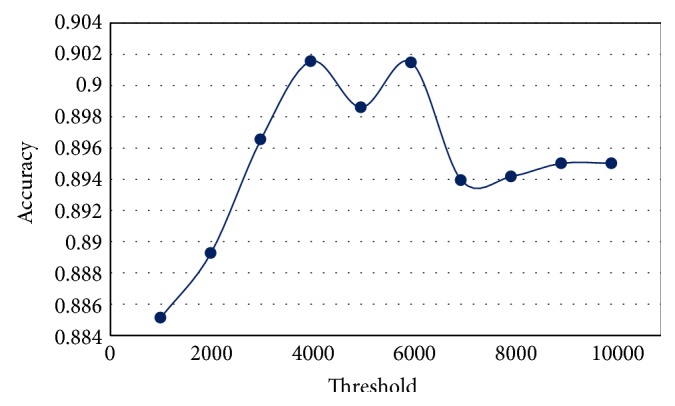
Computing the best threshold value for modified *K*-means.

The modified *K*-means requires a predetermined threshold value to select the initial centroids of clusters. In this experiment, all training datasets in Table 1 are used with testDS1 to compute the average accuracy for different values (1000–10000). The value that yields the highest accuracy is thus chosen as the threshold for modified *K*-means. As can be seen in Figure 2, the threshold value is 4000, as it results in an average of accuracy of 0.90155. We used all the training datasets with only one testing dataset to choose the threshold value because the modified *K*-means approach is applied only on the training dataset to construct the clusters. In all subsequent experiments, the chosen threshold (4000) is employed with hybrid modified *K*-means and C4.5.

### 4.2. MAS-IDS Performance

**Figure 3:**
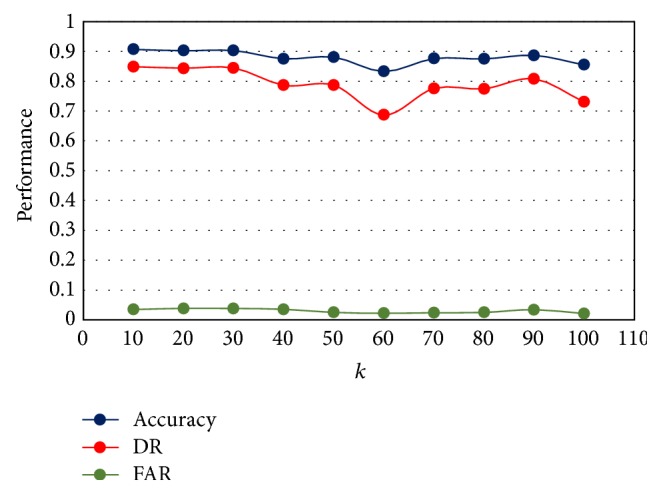
The performance of hybrid standard *K*-means and C4.5.

In order to compare MAS-IDS with the hybrid standard *K*-means and C4.5 [30], the best value of *k* for *K*-means is identified. Typically, *K*-means is run independently for different values of *k* and the partition that appears the most meaningful to the domain expert is selected [62].  Figure 3 shows the performance of hybrid standard *K*-means with C4.5 for different *k* values (*k* = 10,20,30,…, 100). The best *k* value is equal to 10 because it yields the highest accuracy (90.67) and detection rate (84.80). As can be seen, only the false alarm rate percentage (3.46) is not the most optimal, as 2.1 is achieved when *k* = 100. Thus, in all subsequent experiments, we adopt *k* = 10 as the best number of clusters.

**Figure 4:**
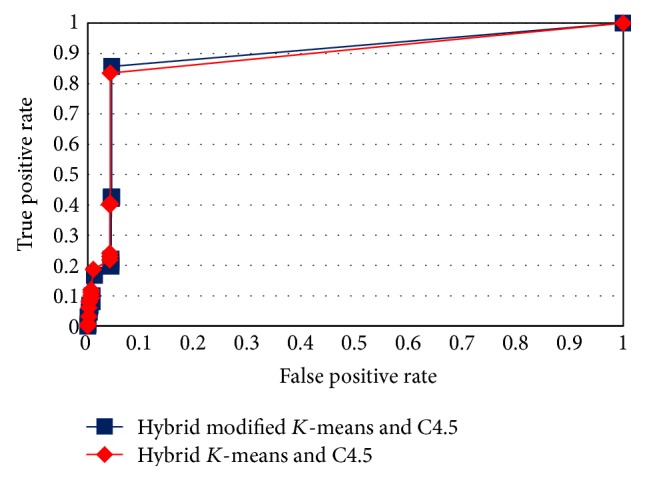
ROC curve for testDS1.

**Figure 5:**
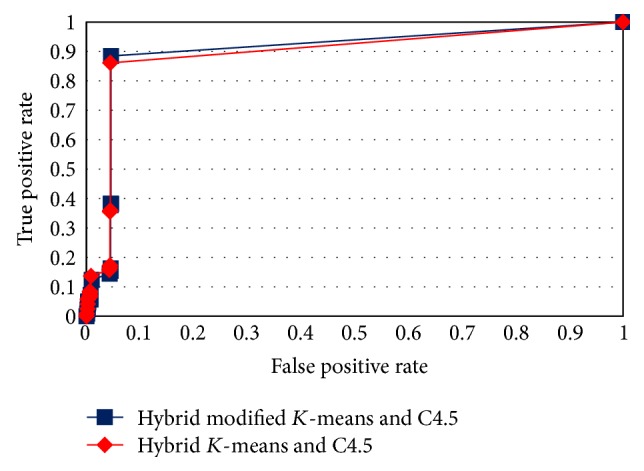
ROC curve for testDS2.

**Figure 6:**
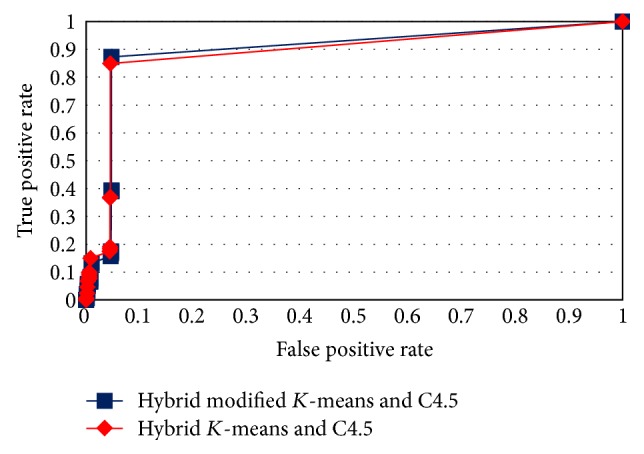
ROC curve for testDS3.

**Figure 7:**
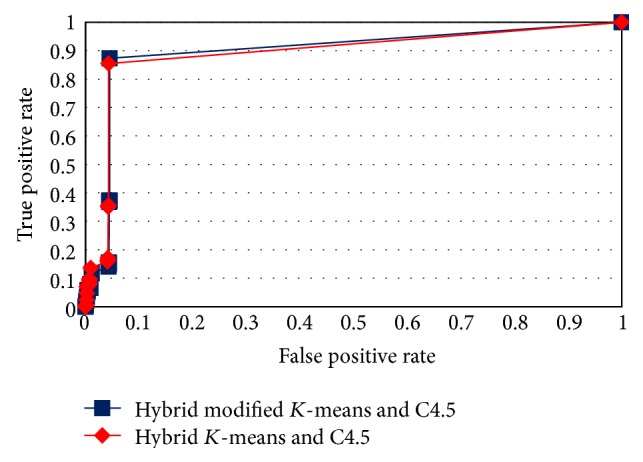
ROC curve for testDS4.

The ROC curves in Figures 4, 5, 6, and 7 show the proposed method performance in comparison with hybrid *K*-means with C4.5 [30].

According to the ROC curves for the proposed hybrid modified *K*-means with C4.5 in MAS-IDS, it achieved better results in comparison with the conventional method. The *t*-test shows that the MAS-IDS significantly improved accuracy, with *p* value < 0.05 (0.00000028). Therefore, the MAS-IDS was tested by computing the classification results pertaining to each training dataset using all testing datasets presented in Table 1.  Table 2 shows the comparison accuracy between MAS-IDS and hybrid *K*-means with C4.5.  Table 3 shows the average results of the MAS-IDS evaluation, along with the comparison with the conventional method and other methods from Weka and Matlab.

**Figure 8:**
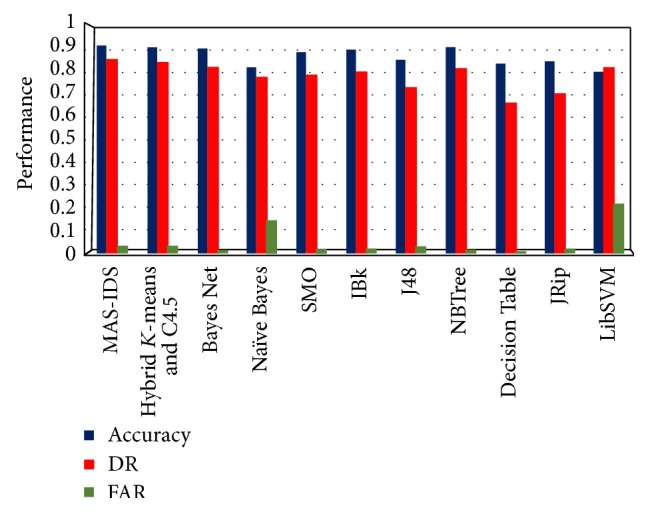
Comparison performance of MAS-IDS with other methods.

As can be seen from Table 3, the MAS-IDS approach achieves higher accuracy and detection rate, as well as *F*-measure. However, the false alarm rate, precision, and specificity are not superior to those achieved by the other methods, especially the Decision Table, which produces the best ratios. In state of the art methods, IDS accuracy is usually measured, due to the equivalence between the error and correct rates. Thus, when comparing various methods, we adopt accuracy as the best measure. On this basis, the performance of our MAS-IDS is superior to other methods, as shown in Table 3. More specifically, the average MAS-IDS accuracy, computed by using all testing datasets and all training datasets, is 0.9113, which is greater than those achieved by other methods.  Figure 8 shows the performance of all methods using data given in Table 3.

### 4.3. MS-IDS Processing Time

The last experiment demonstrates the strength of the MAS-IDS in improving the data classification processing time by using a multiagent system. In this experiment, five of the previously specified computers were used. In addition, we used the forth training dataset (trainDS4) from Table 1 with four new large testing datasets to evaluate the strength of MAS-IDS in processing large datasets in less time.  Table 4 shows the characteristics of the new testing datasets.

To show the ability of the MAS-IDS to reduce the processing time, the MAS-IDS approach is compared with nonagents hybrid modified *K*-means and C4.5. Here, MAS-IDS is implemented every time a new computer is added. In other words, MAS-IDS initially runs on one computer, and when a second computer is added, it starts running on both, and so on until all five computers are used.  Table 5 shows the processing time of this experiment. The maximum number of agents that can be implemented with each computer is eight, because each computer has eight core CPUs and each core can run in parallel only one agent at the time. The training time of this experiment is 88.14 s. It should be noted that the coordinator agent is running on the first computer of the system environment.

**Figure 9:**
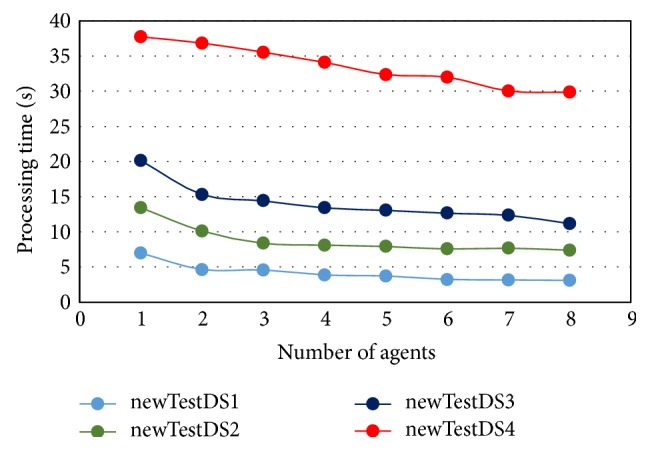
Time required to process the testing datasets in relation to the number of agents.

**Figure 10:**
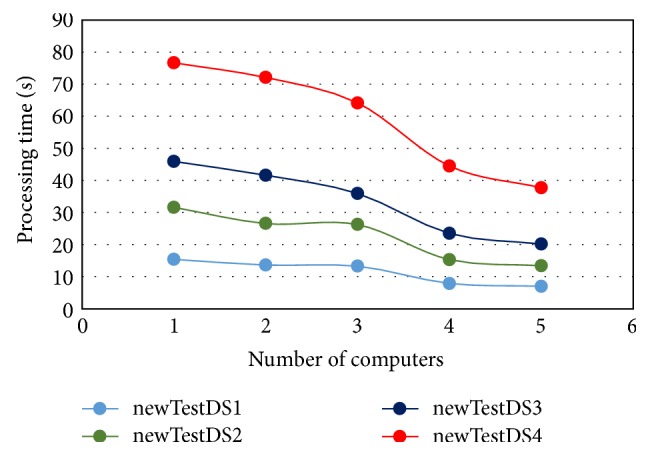
Time required to process the testing datasets in relation to the number of computers.

The results presented in Table 5 are based on the number of computers and the number of agents. The results in the upper left corner of Table 5 pertain to the case of using one computer with one agent. Thus, this is the worst case and should be compared with nonagents hybrid modified *K*-means with C4.5. On the other hand, the results in the lower right corner of Table 4 represent the best case of MAS-IDS (maximum number of computers and agents). Furthermore, as can be seen from the data, when using two computers, due to the cost of data transfer through the network, which will increase the processing time, no improvements are achieved by MAS-IDS relative to other approaches. However, this problem is mitigated by introduction of additional computers. Nonetheless, the MAS-IDS processing time when applied to a large dataset, such as newTestDS4, is inadequate because the dataset subsets are still large and require long time to be transferred to other computers through the network. This problem is eliminated when a large number of computers are employed, due to dividing the dataset into smaller data subsets. Finally, the MAS-IDS processing time decreases with addition of each new computer, as the number of agents also increases. The network specifications, such as bandwidth and speed, play an important role in reducing the MAS-IDS processing time. Figures 9 and 10 show the effect of increasing number of agents and computers on the MAS-IDS processing time, respectively. In Figure 9, the number of computers used with this experiment is five computers, while the number of agents used in experiment of Figure 10 is only one agent as shown in Table 5.

**Figure 11:**
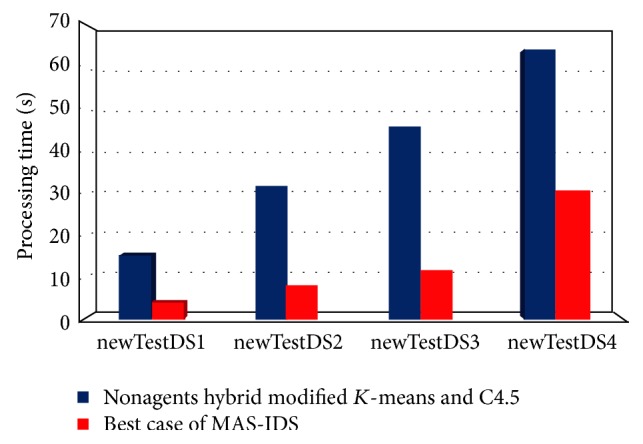
Comparison of MAS-IDS processing time with that of the nonagents hybrid modified *K*-means and C4.5.

The best case of MAS-IDS processing time in comparison with the nonagent hybrid modified *K*-means and C4.5 is shown in Figure 11.

**Figure 12:**
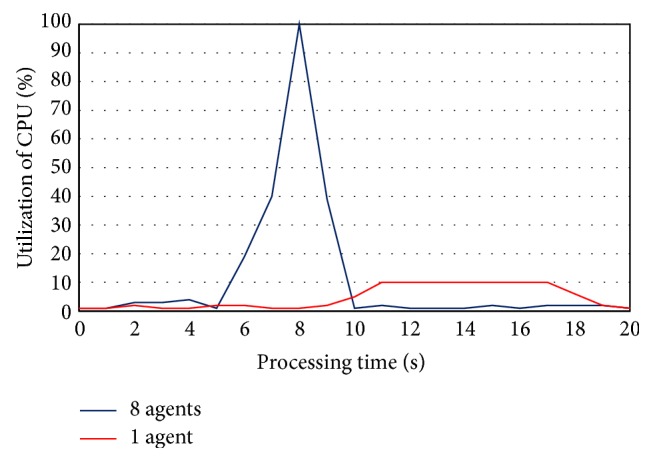
The cost of system resources (CPUs).

Finally, since the proposed system uses each core of CPUs to run one of the analysis agent, then the cost of system resources will be in positive correlation with the increase of the number of agents. At the same time, whenever the number of analysis agent is increasing then the size of subset data analysis will be very small, and thus the analysis process will need only one or two seconds of processing time to achieve it. Consequently, the proposed system makes the balance situation between the physical components (number cores of CPUs) with the number of agents which can be created as (2).  Figure 12 compares the average cost of system resources (consumption of CPUs) when MAS-IDS uses 5 computers with 8 analysis agents at each computer (total 40 agents) and another time when it uses one analysis agent at each computer (total 5 agents) on the same datasets.

From Figure 12, the processing time of the highest peak of utilization of CPU when used one agent (6 sec) is greater than the processing time of the highest peak of utilization of CPU when used 8 agents that consume only one sec. As a consequence, whenever the number of agents is small, the processing time will be long with low cost of system, while whenever the number of agents is increasing, the processing time will be short with high cost of system. The cost of system resources with respect to memory does not exceed 10% in all experiments.

This experiment demonstrates that the MAS-IDS has a great potential to reduce the IDS processing time relative to methods that do not employ agents. The percentage reduction in the processing time for MAS-IDS can reach up to 70% relative to other approaches. In this experiment, we used five computers only. Clearly, with a greater number of computers, a higher percentage reduction in the processing time could be achieved.

## 5. Conclusion

In this work, we have proposed hybrid modified *K*-means with C4.5 for IDS in MAS environment. Hybrid modified *K*-means with C4.5 is used to improve the classification accuracy, while MAS is used to reduce the processing time of IDS. The modification of *K*-means is based on choosing the initial centroids of clusters that represent all cases of the dataset, allowing the number of clusters *k* to be determined. Three types of agents—coordinator, analysis, and communication agent—are used. KDD Cup 1999 dataset is employed, while JADE platform with five computers is used to implement the proposed method.

MAS-IDS demonstrated that multiagent system has significant potential for reducing the IDS processing time. The percentage reduction in processing time of up to 70% was achieved by MAS-IDS. However, the hybrid modified *K*-means with C4.5 approach performed better than the hybrid *K*-means and C4.5, as well as other techniques available in Weka and Matlab. The *t*-test of accuracy that compared MAS-IDS with the conventional *K*-means and C4.5 method confirmed that the former was superior (with *p* value of 0.00000028). This indicates that the MAS-IDS has high potential to improve the performance of intrusion detection systems.

In the future work, we will attempt to improve the IDS accuracy further by combining the proposed method with other techniques. We will also try to implement our method with other datasets and a real data network to make system more suitable for real environment. We will use the new attacks that are detected by system as unknown attacks to retrain the proposed method as a feedback. In addition, we expect to reduce the IDS processing time when using a greater number of computers.

## Figures and Tables

**Pseudocode 1:**
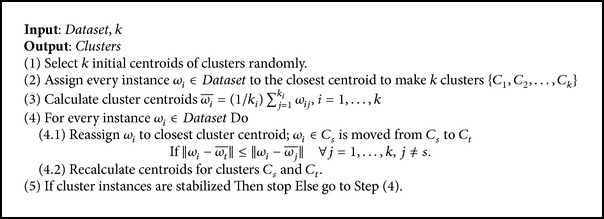
Pseudocode of the standard *K*-means algorithm.

**Pseudocode 2:**
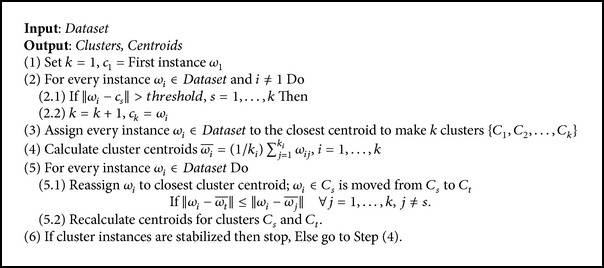
Pseudocode of the modified *K*-means algorithm.

**Pseudocode 3:**
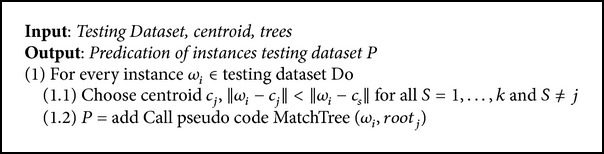
Pseudocode of the determination the closest centroid for an instance.

**Pseudocode 4:**
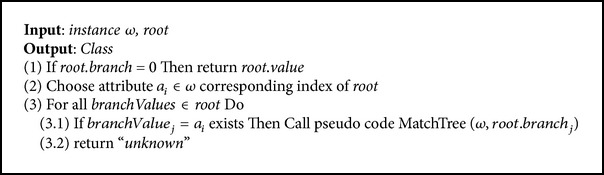
Pseudocode of MatchTree.

**Table 1:** The details of evaluation datasets.

Dataset	Normal	DoS	Probe	R2L	U2R	Total
trainDS1	900	1000	300	500	300	3000
trainDS2	1100	1300	300	800	500	4000
trainDS3	1500	1800	400	1000	300	5000
trainDS4	1800	1800	500	1100	800	6000
testDS1	5000	3000	700	900	400	10000
testDS2	10000	7000	1000	1500	500	20000
testDS3	15000	10000	1500	2500	1000	30000
testDS4	20000	14000	1500	3000	1500	40000

**Table 2:** The accuracy results of the MAS-IDS versus hybrid *K*-means and C4.5 [30].

Training dataset	Testing dataset	MAS-IDS	Hybrid *K*-means and C4.5
trainDS1	testDS1	**0.9031**	0.8993
	testDS2	**0.91745**	0.916021
	testDS3	**0.9126**	0.909633
	testDS4	**0.9147**	0.911625

trainDS2	testDS1	**0.8874**	0.8742
	testDS2	**0.9107**	0.9008
	testDS3	**0.904367**	0.894967
	testDS4	**0.90545**	0.89475

trainDS3	testDS1	**0.9058**	0.8932
	testDS2	**0.92225**	0.9135
	testDS3	**0.91667**	0.9049
	testDS4	**0.916125**	0.90805

trainDS4	testDS1	**0.9099**	0.8963
	testDS2	**0.9215**	0.90815
	testDS3	**0.9155**	0.9016
	testDS4	**0.918025**	0.90625

*p* value		0.00000028	

**Table 3:** Comparison of the MAS-IDS performance with other methods using different measures.

Method	Accuracy	DR	FAR	Precision	Specificity	*F*-measure
MAS-IDS	**0.9113**	**0.8526**	0.0299	0.9665	0.9701	**0.9056**
Hybrid *K*-means and C4.5 (2012)	0.9021	0.8394	0.0353	0.9600	0.9647	0.8954
Bayes Net	0.9017	0.8177	0.0142	0.9829	0.9858	0.8926
Naïve Bayes	0.8150	0.7727	0.1427	0.8578	0.8573	0.8076
SMO	0.8805	0.7785	0.0174	0.9781	0.9826	0.8664
IBk	0.8886	0.7962	0.0190	0.9766	0.9810	0.8771
J48	0.8513	0.7298	0.0273	0.9638	0.9727	0.8299
NBTree	0.9007	0.8096	0.0081	0.9900	0.9919	0.8903
Decision Table	0.8306	0.6631	**0.0020**	**0.9970**	**0.9980**	0.7956
JRip	0.8377	0.6983	0.0229	0.9682	0.9771	0.8111
LibSVM	0.7964	0.8120	0.2191	0.8169	0.7809	0.8068

**Table 4:** Characteristics of new testing datasets used to evaluate the MAS-IDS processing time.

Dataset	Normal	DoS	Probe	R2L	U2R	Total
newTestDS1	35000	35000	10000	10000	10000	100000
newTestDS2	70000	70000	20000	25000	15000	200000
newTestDS3	100000	100000	30000	50000	20000	300000
newTestDS4	150000	150000	30000	50000	20000	400000

**Table 5:** Comparison of processing time required by MAS-IDS and other nonagents hybrid modified *K*-means and C4.5.

Number of agents	Testing dataset	Number of computers (processing time in seconds)				
		1	2	3	4	5
1	newTestDS1	15.349	13.57	13.219	7.841	6.972
	newTestDS2	31.607	26.554	26.228	15.309	13.424
	newTestDS3	45.893	41.587	35.910	23.462	20.162
	newTestDS4	64.172	76.688	72.101	44.509	37.723

2	newTestDS1	8.852	8.304	7.102	5.843	4.630
	newTestDS2	17.339	17.883	16.310	13.160	10.110
	newTestDS3	26.184	27.500	22.502	16.931	15.313
	newTestDS4	40.226	64.332	49.853	37.263	36.832

3	newTestDS1	8.121	8.242	6.940	5.34	4.575
	newTestDS2	13.472	12.767	11.743	9.957	8.374
	newTestDS3	20.735	22.689	20.186	15.788	14.421
	newTestDS4	32.604	52.819	48.814	36.589	35.536

4	newTestDS1	6.699	6.818	5.891	4.631	3.854
	newTestDS2	11.776	14.209	11.104	9.375	8.116
	newTestDS3	18.613	20.927	17.613	14.490	13.413
	newTestDS4	29.399	51.852	46.811	35.564	34.90

5	newTestDS1	6.146	6.659	5.579	4.555	3.715
	newTestDS2	11.534	13.685	10.580	9.198	7.924
	newTestDS3	17.568	20.662	17.497	14.44	13.62
	newTestDS4	29.349	51.527	43.956	34.521	32.349

6	newTestDS1	6.68	6.601	5.393	4.465	3.224
	newTestDS2	11.318	12.922	10.531	8.980	7.567
	newTestDS3	17.419	20.203	16.938	15.788	12.656
	newTestDS4	28.660	50.67	41.475	32.390	31.989

7	newTestDS1	5.871	6.443	5.272	4.258	3.150
	newTestDS2	11.134	12.787	10.494	8.680	7.685
	newTestDS3	17.223	20.683	16.556	15.178	12.362
	newTestDS4	28.438	45.539	39.859	30.447	30.20

8	newTestDS1	5.481	6.204	4.289	4.84	**3.89**
	newTestDS2	11.011	13.24	9.913	8.369	**7.399**
	newTestDS3	17.150	19.713	16.483	14.81	**11.181**
	newTestDS4	**27.303**	40.39	38.156	30.196	29.851
